# Overexpression of ferroptosis defense enzyme Gpx4 retards motor neuron disease of SOD1G93A mice

**DOI:** 10.1038/s41598-021-92369-8

**Published:** 2021-06-18

**Authors:** Liuji Chen, Ren Na, Kirsten Danae McLane, Cody Sylvester Thompson, Ju Gao, Xinglong Wang, Qitao Ran

**Affiliations:** 1 Department of Cell Systems & Anatomy, University of Texas Health San Antonio, 7703 Floyd Curl Dr., San Antonio, TX 78229 USA; 2 Research Service, South Texas Veterans Health Care System, San Antonio, TX USA; 3 Department of Pharmacology and Experimental Neurosciences, University of Nebraska Medical Center, Omaha, NE USA

**Keywords:** Diseases of the nervous system, Cell death

## Abstract

Degeneration and death of motor neurons in Amyotrophic Lateral Sclerosis (ALS) are associated with increased lipid peroxidation. Lipid peroxidation is the driver of ferroptosis, an iron-dependent oxidative mode of cell death. However, the importance of ferroptosis in motor neuron degeneration of ALS remains unclear. Glutathione peroxidase 4 (Gpx4) is a key enzyme in suppressing ferroptosis by reducing phospholipid hydroperoxides in membranes. To assess the effect of increased protection against ferroptosis on motor neuron disease, we generated SOD1^G93A^GPX4 double transgenic mice by cross-breeding GPX4 transgenic mice with SOD1^G93A^ mice, a widely used ALS mouse model. Compared with control SOD1^G93A^ mice, both male and female SOD1^G93A^GPX4 mice had extended lifespans. SOD1^G93A^GPX4 mice also showed delayed disease onset and increased motor function, which were correlated with ameliorated spinal motor neuron degeneration and reduced lipid peroxidation. Moreover, cell toxicity induced by SOD1^G93A^ was ameliorated by Gpx4 overexpression and by chemical inhibitors of ferroptosis in vitro. We further found that the anti-ferroptosis defense system in spinal cord tissues of symptomatic SOD1^G93A^ mice and sporadic ALS patients might be compromised due to deficiency of Gpx4. Thus, our results suggest that ferroptosis plays a key role in motor neuron degeneration of ALS.

## Introduction

Motor neuron degenerative diseases are neurological disorders triggered by degeneration and death of motor neurons that control essential voluntary muscle activity. The most common motor neuron degenerative disease is Amyotrophic Lateral Sclerosis (ALS), in which motor neuron degeneration and death result in progressive paralysis and eventual death due to respiratory failure^1^. Research over past decades has identified many genetic mutations that predispose to early onset of ALS^2^. In addition, mechanisms such as disrupted RNA processing, mitochondrial dysfunction, protein dyshomeostasis, axonal transport disruption, and inflammation have been linked to the pathogenesis of the disease^3–5^. Despite these advances, there are still no efficacious treatments that can significantly alter the disease trajectory of ALS^6^.

Reactive oxygen species (ROS) and their resultant oxidative damage are increased in ALS patient samples and ALS animal models^7, 8^. Polyunsaturated fatty acids contained in membrane phospholipids are prone to be attacked by ROS, triggering a plethora of redox reactions collectively called lipid peroxidation. Lipid hydroperoxides are a primary product of lipid peroxidation, the presence of which can alter membrane properties and impair functions of membrane-associated proteins. Notably, a high load of phospholipid hydroperoxides in membranes can trigger cell death through ferroptosis, an iron-dependent oxidative mode of cell death^9–11^. The defense system against lipid peroxidation consists of lipid-soluble antioxidants and antioxidant defense enzymes. Glutathione peroxidase 4 (Gpx4) is a selenoprotein glutathione peroxidase that uses glutathione as an electron donor to reduce highly toxic lipid hydroperoxides in membranes to less toxic lipid alcohols^12^. Because of its importance in suppressing hydroperoxide levels in phospholipids, Gpx4 serves as the master regulator of ferroptosis^9, 13^. Notably, accumulating evidence indicates that Gpx4 is an essential enzyme for motor neuron health and survival. For example, conditional ablation of Gpx4 in neurons of adult mice triggered a rapid degeneration of spinal motor neurons, resulting in a savage paralysis that resembles accelerated ALS^14^; consistent with Gpx4’s role as the master regulator of ferroptosis, motor neuron degeneration induced by Gpx4 ablation exhibited characteristics of ferroptosis^14^. The rapid death of motor neurons induced by Gpx4 ablation indicated that spinal motor neurons are particularly vulnerable to ferroptosis if the anti-ferroptosis defense system is compromised.

SOD1^G93A^ is the first SOD1 mutant identified in familial ALS cases. SOD1^G93A^ mice are a widely used ALS model that develops motor neuron degenerative disease. Increased lipid peroxidation was reported in SOD1^G93A^ mice^7^. In this study, to determine the role of ferroptosis in motor neuron degeneration in ALS, we investigated the effect of Gpx4 overexpression on motor neuron disease of SOD1^G93A^ mice. We further determined the importance of ferroptosis in cell toxicity induced by SOD1^G93A^ in vitro and evaluated the status of Gpx4 in SOD1^G93A^ mice and ALS patient samples.

## Results

### SOD1^G93A^GPX4 double transgenic mice had extended lifespans

**Figure 1:**
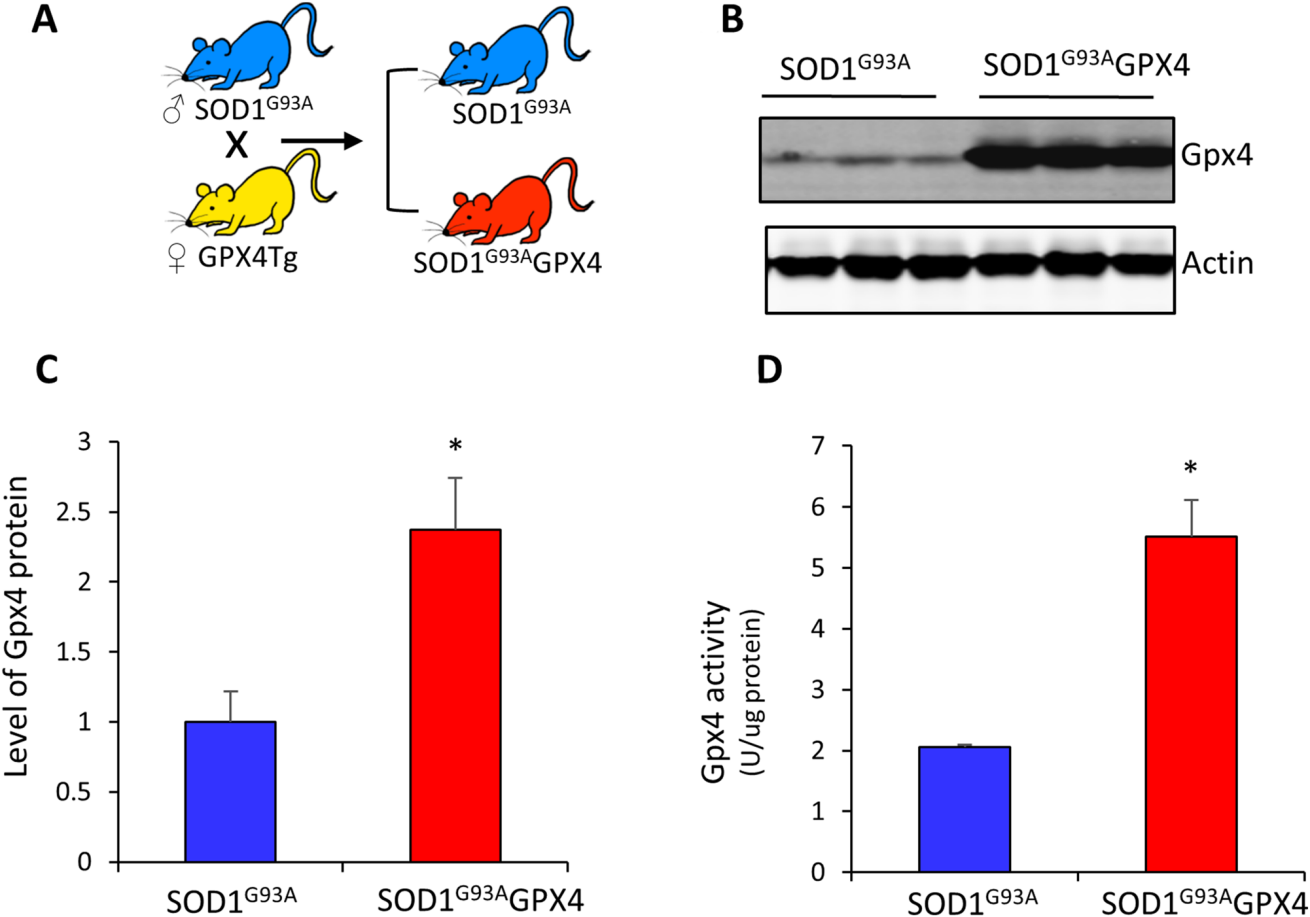
Overexpression of Gpx4 in SOD1^G93A^GPX4 mice. (**A**) Graph of breeding strategy. (**B**) Graph of western blots showing Gpx4 protein in spinal cord tissues of SOD1^G93A^ and SOD1^G93A^GPX4 mice at 60 days of age. (**C**) Quantified results of western blots showing an increased level of Gpx4 protein in SOD1^G93A^GPX4 mice. n = 3 (biological repeats) for both genotypes. (**D**) Gpx4 activities in spinal protein extracts of SOD1^G93A^ mice and SOD1^G93A^GPX4 mice. n = 6 (biological repeats) for both genotypes. *: *p* < 0.05.

SOD1^G93A^ was the first mutation of SOD1 gene identified in familial ALS cases^15^. SOD1^G93A^ mice is a widely used ALS mouse model that exhibits paralytic progression and motor neuron loss similar to those of ALS patients^16^. Gpx4 is the master regulator of ferroptosis due to its critical role in reducing hydroperoxides in phospholipids. To determine whether increased defense against ferroptosis might affect motor neuron disease, we were interested in determining how increased function of Gpx4 might affect lifespan and motor neuron degeneration of SOD1^G93A^ mice. We previously generated a GPX4 transgenic mouse in which the expression of human Gpx4 is directed by an endogenous GPX4 promotor^17^. Overexpression of Gpx4 is observed in many cell types, including neurons, in GPX4 transgenic mice^17, 18^. To generate SOD1^G93A^ mice with enhanced function of Gpx4, we cross-bred female GPX4 transgenic mice with male SOD1^G93A^ mice to obtain offspring control SOD1^G93A^ mice and SOD1^G93A^GPX4 double transgenic mice (Fig. 1A). To verify Gpx4 overexpression in SOD1^G93A^GPX4 mice, we compared levels of Gpx4 protein in spinal cord tissues from SOD1^G93A^GPX4 and control SOD1^G93A^ mice. As shown in Fig. 1B,C, SOD1^G93A^GPX4 mice had a significantly elevated level of Gpx4 protein in spinal cord tissues measured by western blots. We also compared Gpx4 activity in spinal cord tissues of SOD1^G93A^GPX4 and SOD1^G93A^ mice. Spinal protein extracts were obtained, and specific activities of Gpx4 were measured by a Gpx4 activity assay that used phosphatidylcholine hydroperoxide as substrate. Consistent with western blot results, spinal protein extracts of SOD1^G93A^GPX4 mice had a 2.5-fold increase in Gpx4 activity over that of control SOD1^G93A^ mice (Fig. 1D).

**Figure 2:**
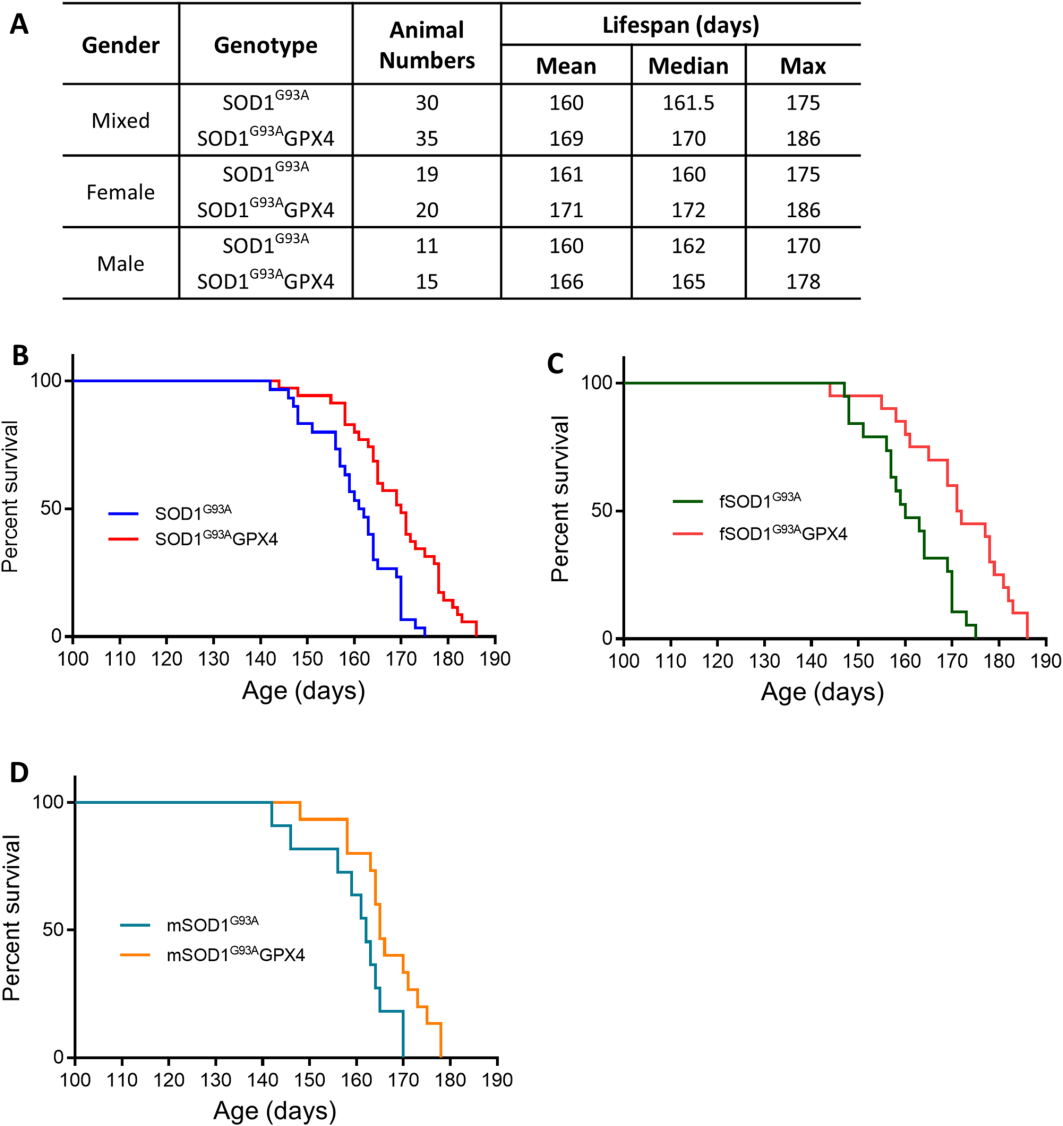
Lifespans and survival curves of SOD1^G93A^ and SOD1^G93A^GPX4 mice. (**A**) Data table indicating lifespans of mixed gender as well as female and male SOD1^G93A^ and SOD1^G93A^GPX4 mice. (**B**) Survival curves of SOD1^G93A^ and SOD1^G93A^GPX4 mice (mixed gender). *p* < 0.0001, The log rank test. (**C**) Survival curves of female SOD1^G93A^ and SOD1^G93A^GPX4 mice. *p* < 0.0005. (**D**) Survival curves of male SOD1^G93A^ and SOD1^G93A^GPX4 mice. *p* < 0.0257.

To determine how Gpx4 overexpression might affect motor neuron disease triggered by SOD1^G93A^, we established cohorts of SOD1^G93A^GPX4 and control SOD1^G93A^ mice and then proceeded to measure their lifespans. In the lifespan study, a mouse would be marked as reaching endpoint and euthanized if it could not right itself within 20 s when placed on its side. The lifespan data of SOD1^G93A^GPX4 mice and control SOD1^G93A^ mice are presented in Fig. 2A. The mixed-gender SOD1^G93A^GPX4 mice (n = 35) had mean, median and maximum lifespans of 169, 170, and 186 days respectively, while the mixed-gender SOD1^G93A^ mice (n = 30) had mean, median and maximum lifespans of 160, 161.5 and 175 days respectively. Figure 2B shows the Kaplan–Meier survival curves of mixed-gender SOD1^G93A^GPX4 and SOD1^G93A^ mice. The log rank test analysis indicated that the difference between the survival curves is highly significant (*p* < 0.0001). Breaking down the data by gender, the mean, median and maximum lifespans of female SOD1^G93A^GPX4 mice (n = 20) were 171, 172, and 186 days respectively, while the mean, median and maximum lifespans of female SOD1^G93A^ mice (n = 19) were 161, 160 and 175 days respectively. And the mean, median and maximum lifespans of male SOD1^G93A^GPX4 mice (n = 15) were 166, 165, and 178 days respectively, while the mean, median and maximum lifespans of male SOD1^G93A^GPX4 mice (n = 11) were 160, 162, and 170 days respectively. The Kaplan–Meier survival curves of female SOD1^G93A^GPX4 mice and SOD1^G93A^ mice are depicted in Fig. 2C, and the difference between the curves is judged to be highly significant (*p* < 0.0005, The log rank test). The Kaplan–Meier survival curves of male SOD1^G93A^GPX4 mice and male SOD1^G93A^ mice (Fig. 2D) are also deemed to be significantly different (*p* = 0.0257). Thus, overexpression of Gpx4 extended the overall survival of SOD1^G93A^GPX4 mice in both genders.

### Delayed disease onset and delayed loss of motor function in SOD1^G93A^GPX4 double transgenic mice

**Figure 3:**
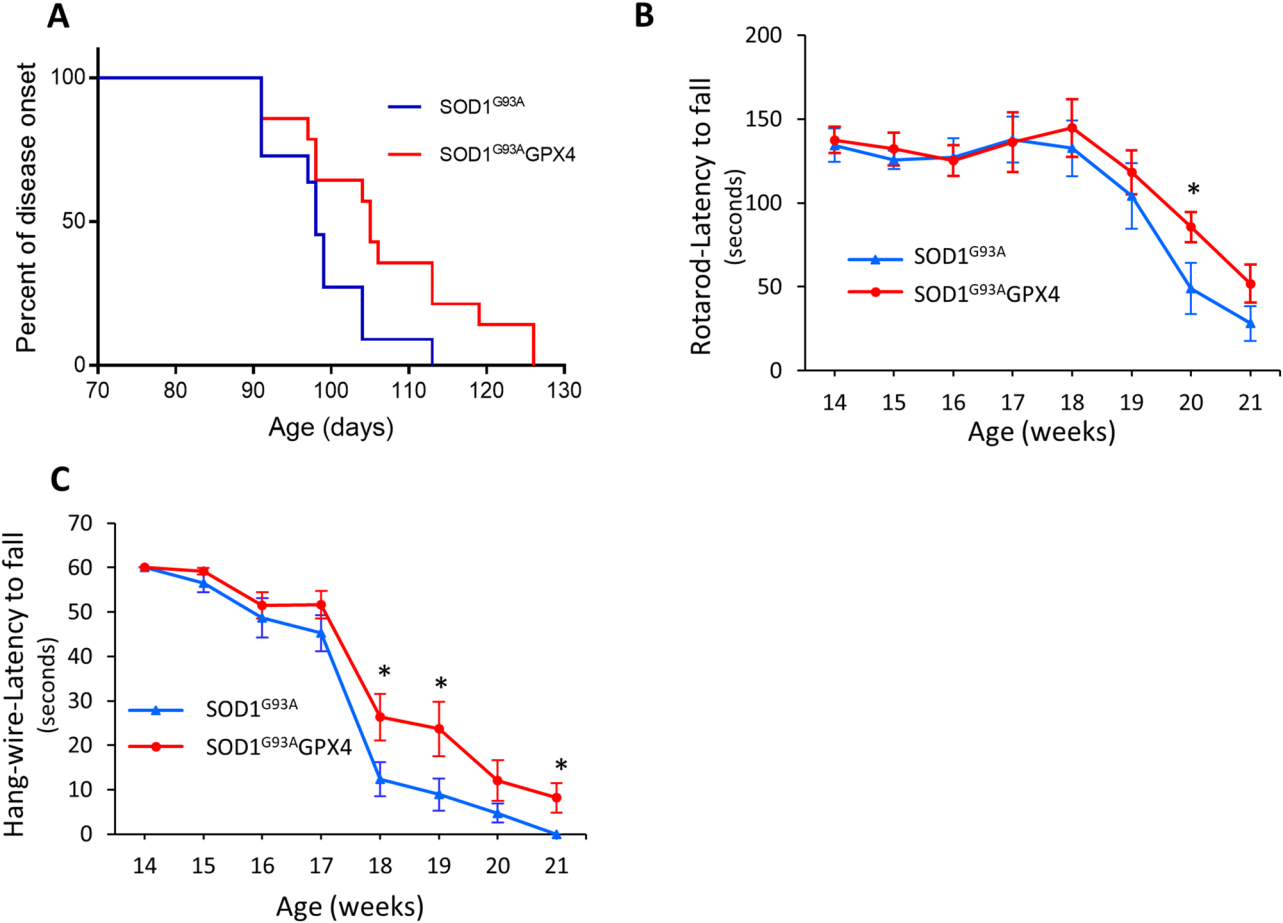
Delayed disease onset and increased locomotor function of SOD1^G93A^GPX4 mice. (**A**) Graph depicting age-associated percentages of disease onset ages for control SOD1^G93A^ and SOD1^G93A^GPX4 mice. *p* < 0.05, The log rank test. (**B**) Performances of control SOD1^G93A^ and SOD1^G93A^GPX4 mice in a rotarod task. (**C**) Performances of control SOD1^G93A^ and SOD1^G93A^GPX4 mice in a hang wire task. n = 11 for SOD1^G93A^ and 14 for SOD1^G93A^GPX4. *: *p* < 0.05.

We next compared disease onset time and locomotor function between control SOD1^G93A^ and SOD1^G93A^GPX4 mice. To determine the time of disease onset, mice were evaluated using the neurological scoring system developed by ALSTDI^19^, and the age when a mouse showed symptoms fitting with a score of 1 was designated as the time of disease onset. The percentages of SOD1^G93A^ and SOD1^G93A^GPX4 mice that had onset of disease are depicted in hazard curves in Fig. 3A. The median ages of disease onset for SOD1^G93A^ and SOD1^G93A^GPX4 mice were 98 days and 105 days, respectively, representing a delay of 7 days in onset time for SOD1^G93A^GPX4 mice. The difference in hazard rates between the two onset time curves was statistically significant as judged by The log rank test (*p* < 0.05), indicating that SOD1^G93A^GPX4 mice had delayed disease onset. To assess locomotor function, the motor coordination function of SOD1^G93A^ and SOD1^G93A^GPX4 mice were measured by a rotarod task, while muscle strength of mice was measured by a hang-wire test. SOD1^G93A^GPX4 mice showed increased rotarod performance starting in week# 18, and the difference between SOD1^G93A^ and SOD1^G93A^GPX4 mice reached statistical significance in week# 20 (Fig. 3B). Compared with control SOD1^G93A^ mice, SOD1^G93A^GPX4 mice also had significantly improved hang-wire performance starting at the age of 18 weeks (Fig. 3C). Thus, SOD1^G93A^GPX4 mice showed improved locomotor function compared with control SOD1^G93A^ mice.

### Ameliorated motor neuron degeneration in SOD1^G93A^GPX4 double transgenic mice

**Figure 4:**
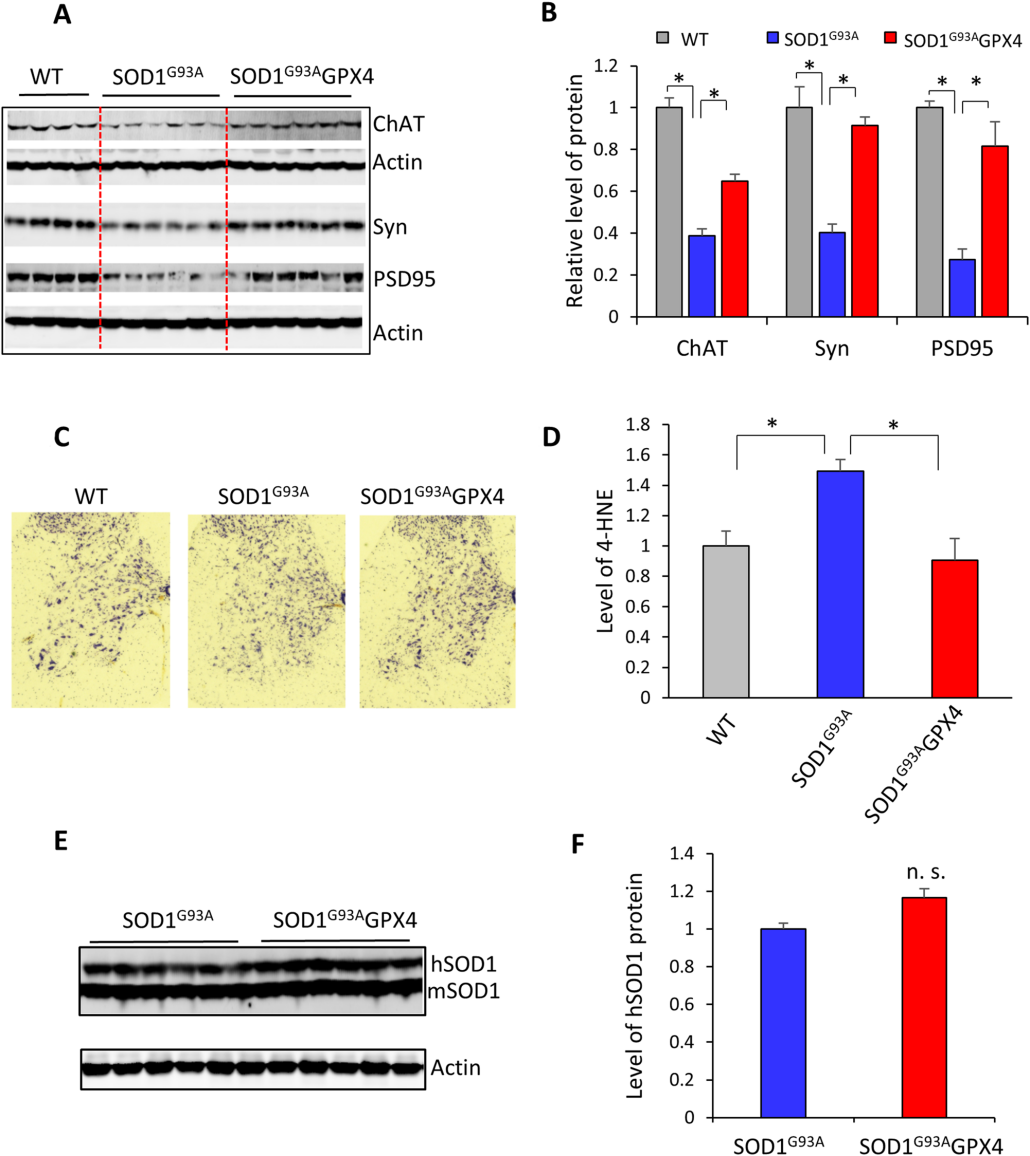
Ameliorated motor neuron degeneration in SOD1^G93A^GPX4 mice. (**A**) Graph of western blot results comparing levels of neuronal marker proteins in lumbar spinal cord tissues of WT, SOD1^G93A^, and SOD1^G93A^GPX4 mice. Syn: synaptophysin. Ages of mice: 120 days. (**B**) Quantified results of western blots. n = 4 for WT and 6 for both SOD1^G93A^ and SOD1^G93A^GPX4. *: *p* < 0.05. (**C**) Images of Nissl-stained lumbar spinal cord sections. (**D)** Levels of 4-HNE protein adducts in lumbar spinal cord tissues of WT, SOD1^G93A^ and SOD1^G93A^GPX4 mice. n = 3 for WT and 4 for both SOD1^G93A^ and SOD1^G93A^GPX4. *: *p* < 0.05. (**E**) Graph of western blots showing levels of hSOD1 and mSOD1 proteins in the lumbar spinal cord tissues of SOD1^G93A^ and SOD1^G93A^GPX4 mice at 120 days of age. (**F**) Quantified result of hSOD1 protein levels. n.s.: not statistically significant.

We next compared motor neuron degeneration between SOD1^G93A^ and SOD1^G93A^GPX4 mice. The advanced stage of paralysis of SOD1^G93A^ mice (150 days of age) was strongly associated with significantly decreased levels of neuronal proteins in lumbar spinal cord tissues, indicating that levels of neuronal proteins are a good indicator of motor neuron degeneration (data shown in Supplementary Material). Therefore, to assess the degrees of motor neuron degeneration, we compared levels of neuronal proteins between SOD1^G93A^ and SOD1^G93A^GPX4 mice at 120 days, an age when the majority of SOD1^G93A^ mice exhibited symptoms such as hind limb tremor and loss of locomotor function (Fig. 3). As expected, compared with WT mice, SOD1^G93A^ mice showed a significantly decreased level of ChAT (Choline acetyltransferase), a motor neuron-specific protein (Fig. 4A,B). In addition, SOD1^G93A^ mice also showed significantly decreased levels of synaptophysin, a presynaptic marker protein, and PSD95, a postsynaptic marker protein (Fig. 4A,B). Notably, compared with SOD1^G93A^ mice, SOD1^G93A^GPX4 mice exhibited increased levels of ChAT, synaptophysin, and PSD95. Consistent with the increased levels of neuronal/synaptic marker proteins in SOD1^G93A^GPX4 mice, Nissl staining (Fig. 4C) of spinal cord sections showed increased integrity of spinal motor neurons in SOD1^G93A^GPX4 mice visually compared with SOD1^G93A^ mice. Thus, our data collectively indicate that motor neuron degeneration was attenuated in SOD1^G93A^GPX4 mice.

4-hydroxylnonenal (4-HNE) is a lipid peroxidation end product that is elevated in SOD1^G93A^ mice^7^. To determine whether overexpression of Gpx4 reduced lipid peroxidation, we also compared levels of 4-HNE adducts in spinal proteins from WT, SOD1^G93A^, and SOD1^G93A^GPX4 mice. Consistent with prior findings^7^, spinal proteins from SOD1^G93A^ mice showed an increased level of 4-HNE compared with WT mice (Fig. 4D). Notably, compared with control SOD1^G93A^ mice, SOD1^G93A^GPX4 mice exhibited a decreased level of 4-HNE adducts in spinal proteins. Thus, the attenuated motor neuron degeneration in SOD1^G93A^GPX4 mice was associated with reduced lipid peroxidation.

### No difference in SOD1^G93A^ levels between SOD1^G93A^ mice and SOD1^G93A^GPX4 mice

The motor neuron disease of SOD1^G93A^ mice is driven by expression and accumulation of SOD1^G93A^ protein. To determine whether the extended lifespan and delayed disease onset of SOD1^G93A^GPX4 mice might be caused by alterations in SOD1^G93A^ expression and accumulation, we compared SOD1^G93A^ protein levels in lumbar spinal cord tissues between SOD1^G93A^GPX4 and SOD1^G93A^ mice. The western blots of human SOD1 (hSOD1) and murine SOD1(mSOD1) proteins are shown in Fig. 4E, and the quantified results are presented in Fig. 4F. Compared with SOD1^G93A^ mice, SOD1^G93A^GPX4 mice showed a slightly higher level of SOD1^G93A^ protein, although the difference did not reach statistical significance. No difference in hSOD1 mRNA levels was observed between SOD1^G93A^ and SOD1^G93A^GPX4 mice (data shown in Supplementary Material). Thus, the increased lifespan and delayed disease onset of SOD1^G93A^GPX4 mice were not associated with reduction of SOD1^G93A^ expression and accumulation.

### SOD1^G93A^ toxicity was ameliorated by Gpx4 overexpression and chemical inhibitors of ferroptosis in vitro

**Figure 5:**
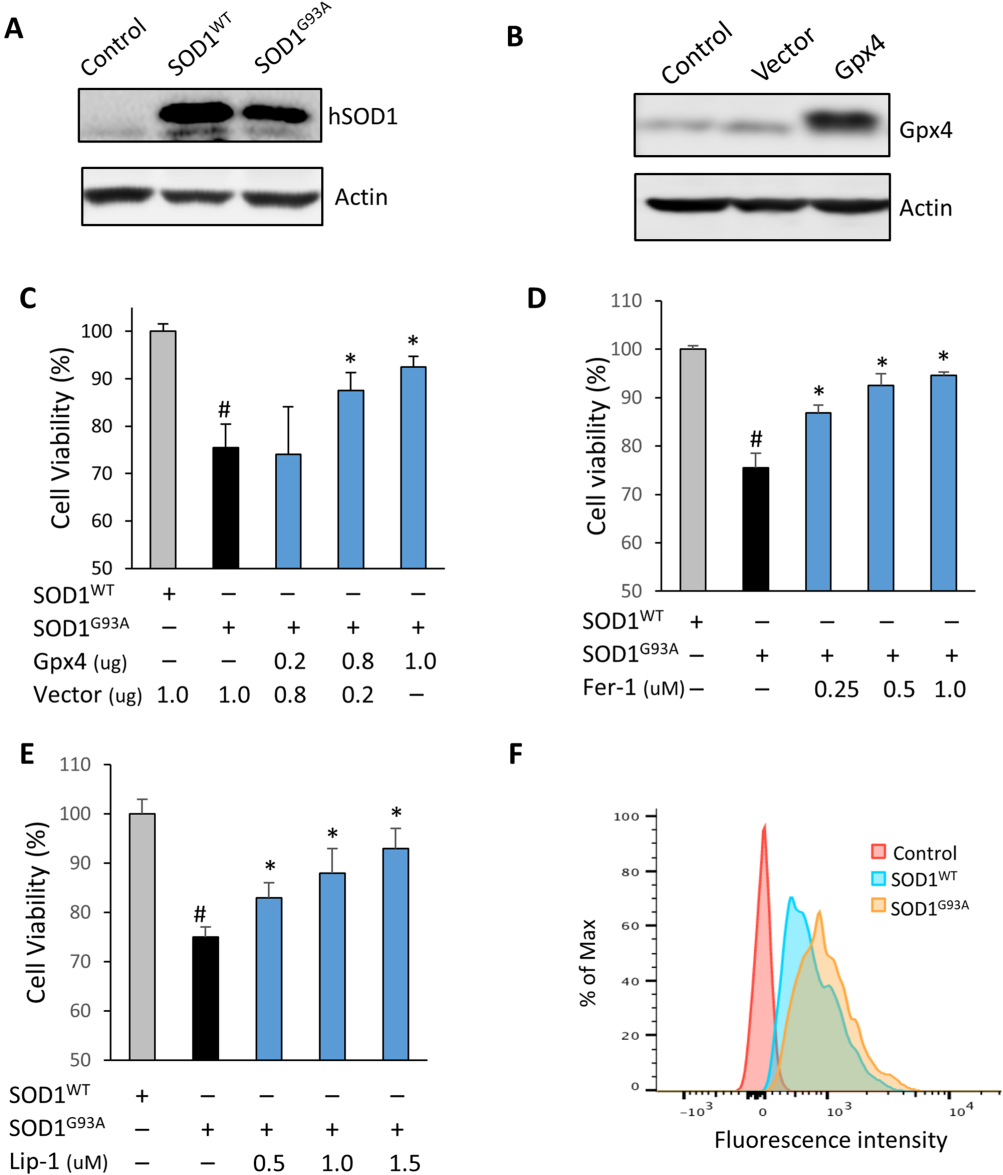
SOD1^G93A^ toxicity was reduced by Gpx4 overexpression and by inhibitors of ferroptosis. (**A**) Graph of Western blots showing levels of human SOD1 protein in NSC-34 motor neuron cells transfected with constructs expressing SOD1^WT^ or SOD1^G93A^. Control cells were un-transfected. (**B**) Graph of western blots showing increased Gpx4 protein in NSC-34 cells transfected with the GPX4 construct. (**C**) Increased survival of NSC-34 cells co-transfected with SOD1^G93A^ construct and Gpx4 construct. (**D**) Fer-1 (ferrostatin-1) treatment increased survival of NSC-34 cells transfected with SOD1^G93A^. (**E**) Lip-1 (liproxstatin-1) treatment increased survival of NSC-34 cells transfected with SOD1^G93A^. . #: *p* < 0.05, SOD1^G93A^ vs SOD1^WT^; *: *p* < 0.05, SOD1^G93A^ with treatment vs SOD1^G93A^ without treatment. (**F**) Representative flow cytometry graph showing oxidation of BODIPY581/591 C11 in control cells and cells transfected with SOD1^WT^ or SOD1^G93A^.

The lifespan extension, delayed disease onset, and delayed loss of motor function of SOD1^G93A^GPX4 mice prompted us to further look at the role of ferroptosis in SOD1^G93A^ toxicity. To that end, we obtained plasmid constructs expressing human wildtype SOD1 (SOD1^WT^) or SOD1^G93A^ that were originally generated by Fisher lab^20^. To confirm the expression of SOD1 variants, NSC-34 cells, a motor neuron-like hybrid cell line, were transfected with SOD1^WT^ or SOD1^G93A^ constructs. As shown in Fig. 5A, NSC-34 cells transfected with SOD1^WT^ or SOD1^G93A^ constructs had increased levels of human SOD1 protein. We also generated a Gpx4 expression construct in which the expression of human Gpx4 is driven by the ubiquitous CAG promoter. As shown in Fig. 5B, transfection with the Gpx4 construct resulted in increased Gpx4 protein in NSC-34 cells. We also established an in vitro SOD1^G93A^ toxicity model, which involved transfection of NSC-34 cells with SOD1^WT^ or SOD1^G93A^ constructs, and then assessed cell viability using a trypan blue exclusion method at 72 h post-transfection. In this model, transfection by SOD1^WT^ had minimum effect on cell survival, but transfection by SOD1^G93A^ caused a moderate but consistent decrease (⁓25%) in cell viability. To determine the effect of Gpx4 overexpression on SOD1^G93A^-induced cell death, NSC-34 cells were transfected with SOD1^G93A^ and vector constructs or SOD1^G93A^ and Gpx4 constructs, and cell viabilities were then measured and compared. As shown in Fig. 5C, compared with cells transfected with SOD1^G93A^ and vector constructs, cells transfected with SOD1^G93A^ and Gpx4 constructs had increased cell viability, indicating that Gpx4 overexpression confers protection against SOD1^G93A^ toxicity in vitro*.*

To determine the role of ferroptosis in SOD1^G93A^ toxicity, we further evaluated the effect of treatment with ferroptosis inhibitors on survival of cells transfected with SOD1^G93A^. Ferrostatin-1 (Fer-1) is a small molecule inhibitor of ferroptosis functioning by scavenging lipid ROS^21^. To determine the effect of Fer-1 treatment on SOD1^G93A^-induced cell death, after transfection with the SOD1^G93A^ construct, cells were immediately treated with increasing doses of Fer-1. As shown in Fig. 5D, Fer-1 treatment significantly increased viability of NSC-34 cells transfected with SOD1^G93A^. Liproxstatin-1 (Lip-1) is another lipid ROS-scavenging ferroptosis inhibitor^21, 22^. As shown in Fig. 5E, treatment with Lip-1 also significantly increased viability of NSC-34 cells transfected with SOD1^G93A^. Thus, consistent with the result of Gpx4 overexpression, treatment with Fer-1 or Lip-1 significantly alleviated cell death induced by SOD1^G93A^.

Lipid ROS is closely associated with ferroptosis, so we were also interested in determining whether SOD1^G93A^ induces lipid ROS. BODIPY581/591 C11 is a lipid ROS sensor^9^. Consistent with its role in lipid ROS sensing, oxidation of BODIPY581/591 C11 was observed in NSC-34 cells treated with Erastin, a known ferroptosis inducer that increases lipid ROS^9^ (data shown in Supplementary Material). To assess the abilities of SOD1^WT^ and SOD1^G93A^ in generating lipid ROS, after transfection with SOD1^WT^ or SOD1^G93A^ construct, cells were loaded with BODIPY581/591 C11 and analyzed by flow cytometry. As shown in Fig. 5F, cells transfected with both SOD1^WT^ and SOD1^G93A^ constructs exhibited increased oxidation of BODIPY581/591 C11. However, compared with cells transfected with SOD1^WT^, cells transfected with SOD1^G93A^ exhibited a mild increase of BODIPY581/591 C11 oxidation which was consistently observed across multiple experiments.

### Compromised anti-ferroptosis defense in SOD1^G93A^ mice and ALS patient samples

**Figure 6:**
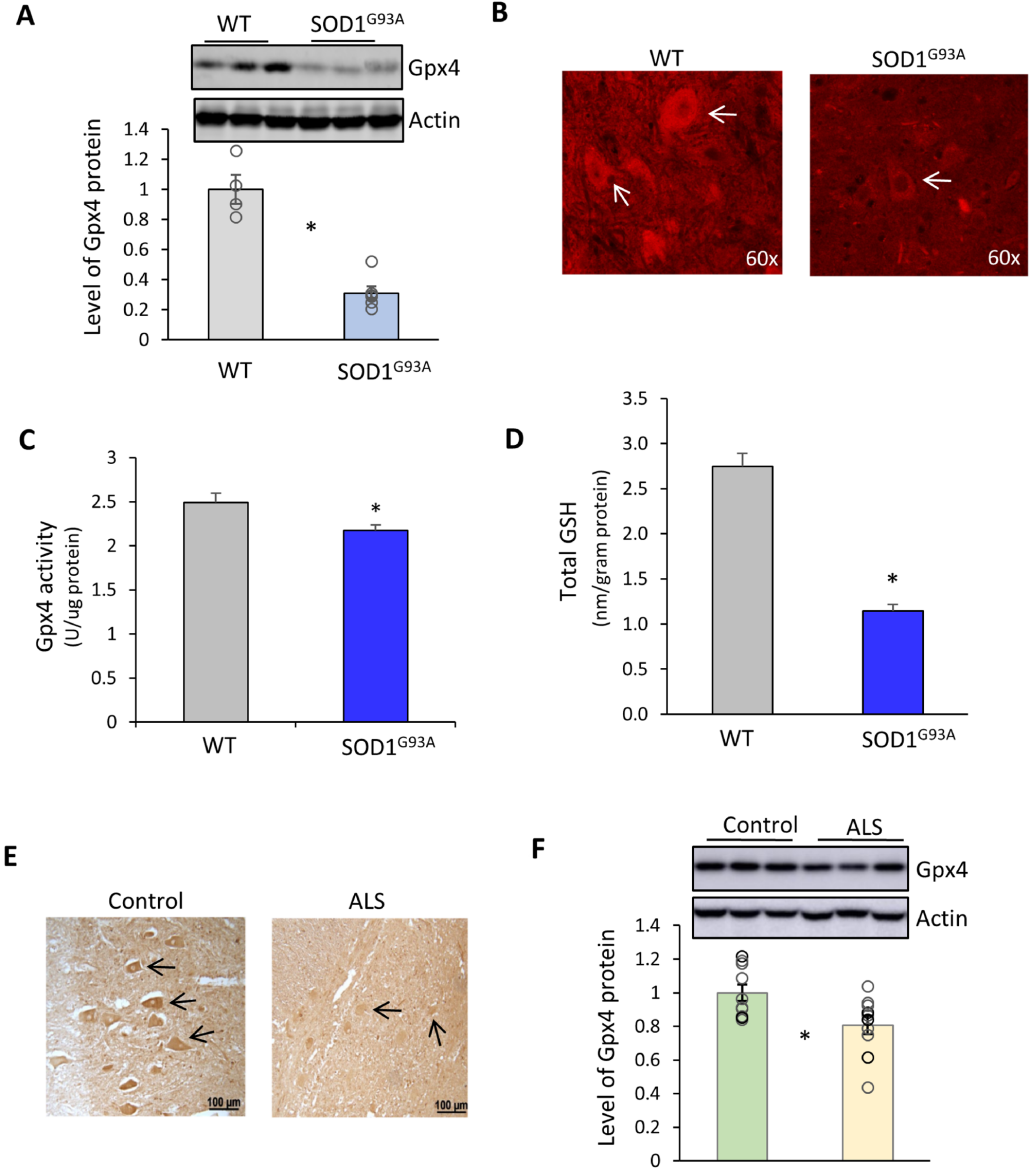
Deficiency of Gpx4 in the spinal cords of symptomatic SOD1^G93A^ mice and of ALS patient samples. (**A**) Graph of western blots showing reduced level of Gpx4 protein in spinal tissues of symptomatic SOD1^G93A^ mice (120 days of age). n = 4 for WT and 6 for SOD1^G93A^. *: *p* < 0 .05. (**B**) Immunofluorescence images of lumbar spinal cord sections from WT and SOD1^G93A^ mice stained with an anti-Gpx4 antibody. Arrow heads indicate motor neurons. (**C**) Gpx4 activities in spinal protein extracts of WT and symptomatic SOD1^G93A^ mice. (**D**) Graph indicating total GSH levels in lumbar spinal cord tissues of WT and symptomatic SOD1^G93A^ mice. n = 4 for WT and 6 for SOD1^G93A^. *: *p* < 0.05. (**E**) Representative images of spinal cord sections of ALS and Control (non-ALS) cases stained immunohistochemically with an anti-Gpx4 antibody. Arrow heads indicate motor neurons. (**F**) Graph of western blots showing reduced levels of Gpx4 protein in spinal tissues from ALS cases compared to Control (non-ALS) cases. n = 10, *: *p* < 0 .05.

Because of Gpx4’s unique role in reducing lipid hydroperoxides to suppress lipid peroxidation and regulate ferroptosis, we were also interested in determining the status of Gpx4 in SOD1^G93A^ mice. To that end, we compared levels of Gpx4 protein in spinal cord tissues obtained from SOD1^G93A^ mice and control WT mice at two ages, an asymptomatic age of 60 days and a symptomatic age of 120 days. Although no difference in Gpx4 protein levels was observed in asymptomatic SOD1^G93A^ mice (data shown in Supplementary Material), symptomatic SOD1^G93A^ mice showed a significant decrease of Gpx4 protein (~ 43% reduction) compared with age-matched WT mice (Fig. 6A). Consistent with western blot results, spinal motor neurons from symptomatic SOD1^G93A^ mice also exhibited decreased intensity of Gpx4 immunofluorescence (Fig. 6B). To determine if deficiency of Gpx4 in symptomatic SOD1^G93A^ mice was due to reduced Gpx4 expression, we compared levels of Gpx4 mRNA between symptomatic SOD1^G93A^ mice and age-matched WT mice, but didn’t detect any decrease in Gpx4 mRNA levels in SOD1^G93A^ mice (data shown in Supplementary Material). Therefore, the deficiency of Gpx4 in symptomatic SOD1^G93A^ mice appeared to have occurred post-transcriptionally. The reduced Gpx4 protein suggests that the function of Gpx4 was compromised in symptomatic SOD1^G93A^ mice. To corroborate this, we measured the activity of Gpx4 in spinal protein extracts. As shown in Fig. 6C, spinal protein extracts from symptomatic SOD1^G93A^ mice showed decreased Gpx4 activity compared to those from WT mice. Although the extents of decreases varied between the assays, an expected outcome due to differences in underlying technologies, the results were in agreement that a deficiency of Gpx4 occurred in symptomatic SOD1^G93A^ mice. Gpx4 uses glutathione as hydrogen donor to reduce lipid hydroperoxides, so we also determined the level of GSH in spinal cord tissues. As shown in Fig. 6E, decreased total GSH was observed in SOD1^G93A^ mice as well. The deficiency of Gpx4 and reduced level of GSH indicated that the anti-ferroptosis defense was compromised in symptomatic SOD1^G93A^ mice.

Our findings that symptomatic SOD1^G93A^ mice had a deficiency of Gpx4 prompted us to further investigate the status of Gpx4 in ALS patients. To look at the status of Gpx4 in ALS patients, we compared levels of Gpx4 protein in lumbar spinal cord tissues from a cohort of ALS patients and a cohort of control patients. Each cohort consisted of 10 patient cases, and the ALS cases were classified as sporadic cases with no known genetic mutations. The case information such as sex and age are presented in Supplementary Material. We analyzed spinal cord sections from ALS cases and Control cases by immunohistochemistry with an anti-Gpx4 antibody. As shown by representative images in Fig. 6E, motor neurons in the Control section exhibited a whole-cell staining pattern that was consistent with the multi-subcellular localization nature of Gpx4. Notably, in the ALS section, the remaining spinal motor neurons showed weak staining of Gpx4. To assess Gpx4 level quantitatively, protein extracts of lumbar spinal cord tissues were obtained and then probed with an anti-Gpx4 antibody. As shown in Fig. 6F, the mean Gpx4 protein level of the ALS patient group was 80.7% of that of the control patient group, representing a modest but statistically significant decrease (⁓19% reduction, *p* = 0.018, Student’s *t*-test). Thus, these results indicated that the cohort of sporadic ALS patients had a deficiency of Gpx4 protein.

## Discussion

The motor neuron protective role of Gpx4 was previously reported in WT mice^14^. In this study, we determined whether enhanced function of Gpx4 could confer protection to motor neurons of SOD1^G93A^ mice. We showed that SOD1^G93A^GPX4 mice had significantly extended lifespans compared with control SOD1^G93A^ mice. Lifespan extension conferred by Gpx4 overexpression was not gender-specific, as both male and female SOD1^G93A^GPX4 mice had increased lifespans compared with gender-matched control SOD1^G93A^ mice. SOD1^G93A^GPX4 mice also showed delayed disease onset and increased locomotor function that were correlated with ameliorated motor neuron degeneration. These results demonstrated that enhanced function of Gpx4 exerted a protective role in retarding motor neuron disease of SOD1^G93A^ mice. The extent of lifespan extension in SOD1^G93A^GPX4 mice looks modest, as the mean lifespans of male and female SOD1^G93A^GPX4 mice were respectively increased by 6 and 10 days, while the maximum lifespans of male and female SOD1^G93A^GPX4 mice were respectively increased by 8 and 11 days. However, SOD1^G93A^ mice are an ALS model in which the onset and progression of motor neuron disease are driven by expression and accumulation of SOD1^G93A^ protein. As such, interventions aimed to reduce SOD1^G93A^ expression and/or SOD1^G93A^ protein accumulation are most likely to achieve large rescuing effects in this model, whereas interventions that do not directly target the SOD1^G93A^ mechanisms are less likely to achieve success in retarding motor neuron disease of this model^23–25^. We showed that overexpression of Gpx4 did not alter the dominant driving force of motor neuron disease in SOD1^G93A^ mice, as there were no differences in levels of SOD1^G93A^ message and SOD1^G93A^ protein between SOD1^G93A^ and SOD1^G93A^GPX4 mice. As such, the modest lifespan extension of SOD1^G93A^GPX4 mice is much more impressive than it appears.

Increased lipid peroxidation has been reported in ALS patients and ALS mouse models^7, 26–28^, suggesting that ferroptosis may be involved in the pathogenesis of ALS. Recently, through evaluating blood-based prognostic indicators in ALS patients, Devos et al. identified four biomarkers with predictive potential for disease progression that were closely associated with ferroptosis^28^. Edaravone (3-methyl-1-phenyl-2-pyrazolin-5-one) is a free radical scavenger approved for the treatment of patients with ALS^6^, the beneficial effect of which has been suggested to be due to its ability to suppress ferroptosis^29, 30^. Previous studies through chelating iron and altering GSH level also suggest the involvement of ferroptosis in the pathogenesis of SOD1^G93A^ mice^31–33^. In this study, we showed that the degeneration of motor neurons was associated with increased lipid peroxidation in SOD1^G93A^ mice, and that the extended lifespans, delayed disease onset, and increased locomotor function of SOD1^G93A^GPX4 mice were concomitantly associated with reduced lipid peroxidation. The importance of lipid peroxidation in motor neuron degeneration of SOD1^G93A^ mice was further supported by different outcomes observed between SOD1^G93A^ mice with overexpression of Gpx4 or Gpx1. Gpx1 is another prominent member of the glutathione peroxidase family. While both Gpx1 and Gpx4 can detoxify H_2_O_2_ and fatty acid hydroperoxides, unlike Gpx4, Gpx1 does not have the ability to reduce hydroperoxides in membrane lipids^34^. Previously, it was reported that SOD1^G93A^ mice with elevated brain Gpx1 activity had no increase in lifespan and no delay in onset of disease^35^. The beneficial outcome conferred by Gpx4 overexpression thus accentuates the importance of lipid hydroperoxides in motor neuron degeneration of SOD1^G93A^ mice. Because high levels of phospholipid hydroperoxides promote ferroptosis^10^, our results from SOD1^G93A^GPX4 mice thus support the notion that ferroptosis is a key mode of cell death in motor neuron degeneration of SOD1^G93A^ mice.

We also assessed the role of ferroptosis in SOD1^G93A^ toxicity in vitro. We showed that SOD1^G93A^-induced death of NSC-34 cells was ameliorated by Gpx4 overexpression as well as by treatment with Fer-1 and Lip-1, two small molecule inhibitors of ferroptosis. Moreover, compared to SOD1^WT^, SOD1^G93A^ also elicited an increased level of lipid ROS in transfected cells. Our results thus indicate that SOD1^G93A^ is capable of inducing ferroptosis. However, the mechanisms of ferroptotic cell death induced by SOD1^G93A^ are unclear. SOD1^G93A^ was reported to have increased hydrophobicity^36^, which could lead to increased lipid ROS generation. SOD1^G93A^ could also impair the ferroptosis defense system as shown in tissues of symptomatic SOD1^G93A^ mice. The contributions of these mechanisms to ferroptotic cell death will need to be illustrated in future studies. We are cognizant that SOD1^G93A^ has been shown previously to induce apoptosis^37–39^. At present, it is unclear how different modalities of cell death are triggered in cells that are subject to stressful conditions such as increased lipid ROS. It is possible that the specific mode of cell death (e.g., apoptosis or ferroptosis) may be dependent on the severity and duration of stress as well as the redox state of the cells. In our cell model, SOD1^G93A^ induced only modest toxicity to NSC-34 cells, which may be the reason why we found ferroptosis was largely responsible for death induced by SOD1^G93A^ in this model. In SOD1^G93A^ mice, the facets of ferroptosis in motor neuron degeneration, such as when it started and to what extent it was responsible for the overall cell death occurred, remain unclear. At present, the characterization of ferroptosis is hindered by a dearth of specific markers of ferroptosis for in vivo use. Therefore, future research on the dynamics of ferroptosis in motor neuron degeneration of ALS models such as SOD1^G93A^ mice is clearly warranted.

In this study, we further showed that symptomatic SOD1^G93A^ mice had a deficiency of Gpx4 in spinal cord tissues. Symptomatic SOD1^G93A^ mice also showed a reduced level of GSH. GSH is the reductant that Gpx4 uses to reduce lipid hydroperoxides. The reduced Gpx4 protein and decreased GSH level indicated that motor neurons had decreased capacity to reduce lipid hydroperoxides in symptomatic SOD1^G93A^ mice. The increased lipid ROS brought about by SOD1^G93A^ protein coupled with a compromised anti-ferroptosis defense system would render motor neurons extremely vulnerable to ferroptosis. Interestingly, spinal tissues from a cohort of sporadic ALS cases also showed a reduced level of Gpx4 protein. The deficiency of Gpx4 is unlikely to be attributed to the slight difference in ages between the ALS cases and Control cases. To our knowledge, this study is the first to report a deficiency of Gpx4 in ALS patient samples. Our results suggest that spinal motor neurons of ALS patients have suboptimal protection against ferroptosis and that deficiency of Gpx4 may be a contributing factor to motor neuron degeneration in at least some ALS patients. If confirmed in other cohorts of ALS patients, deficiency of Gpx4 could represent a novel vulnerability of motor neurons in ALS.

At present, the causes of Gpx4 deficiency are unclear. In symptomatic SOD1^G93A^ mice, because no decrease in Gpx4 mRNA level was observed, the deficiency of Gpx4 likely occurred at the post-transcriptional level. Recently, the homeostasis of Gpx4 protein was reported to be governed by chaperone-mediated autophagy^40^. Dysfunctions of protein quality control systems such as the autophagy-lysosomal system are well demonstrated in SOD1^G93A^ mice and ALS patients^41, 42^. Therefore, it will be interesting to determine whether impaired autophagy-lysosomal systems may be responsible for Gpx4 deficiency in symptomatic SOD1^G93A^ mice and ALS patients in future studies.

In conclusion, our results from this study indicate that enhanced function of Gpx4 retards motor neuron disease of SOD1^G93A^ mice, likely through a mechanism of suppressing ferroptosis of motor neurons. Our results highlight the importance of ferroptosis as a potential target of intervention for ALS therapeutics development.

## Methods

### Animals and animal procedures

SOD1^G93A^ mice (in C57BL/6 J background) [B6SJL-Tg(SOD1*G93A)1Gur/J] were obtained from Jackson Laboratory. Male SOD1^G93A^ mice were cross-bred with female GPX4 transgenic mice (Tg 5 line, in C57BL/6 J background)^17^, and offspring SOD1^G93A^ and SOD1^G93A^GPX4 mice were randomly enrolled in longitudinal cohorts and cross-sectional cohorts. Mice were housed in standard rodent microisolator cages with filtered tops, with 2–4 animals per cage. Mice were housed under a 12/12-h light/dark cycle with ad libitum access to food and water. Mice were checked twice daily starting at week 7. For lifespan determination, a mouse would be marked as reaching endpoint and euthanized if it could not right itself within 20 s when placed on its side. The studies were conducted in accordance with recommendations by Scot et al.^43^.

To determine the onset of disease, mice were evaluated daily using the neurological scoring system developed by ALSTDI that employs a scale of 0 to 4^19^. The age when a mouse reached the score of 1 (i.e., collapse or partial collapse of leg extension towards lateral midline (weakness) or trembling of hind legs during tail suspension) was designated as the time of disease onset.

Rotarod performance was measured with a Rotamex 4/8 (Columbus Instruments, Columbus, OH) using an accelerating rod protocol. The initial speed of the rod was set to 2 rpm with a linear acceleration to 20 rpm over 300 s. The latency to fall was measured in three trials. The mean latency was registered and used as an indicator of rotarod performance.

A hang wire test was used to evaluate muscle strength. The mouse was placed on the bottom of a wire mesh basket (12″ × 8″ × 4″), which was then inverted and suspended above the home cage. The latency to fall was recorded. The mean latency of three trials was used as an indicator of hang wire performance.

Procedures for handling mice in this study were reviewed and approved by the Institutional Animal Care and Use Committees of the University of Texas Health San Antonio and the Audie Murphy Memorial Veterans Hospital, South Texas Veterans Health Care System.

The study was carried out in compliance with the ARRIVE guidelines. All methods were performed in accordance with the relevant guidelines and regulations.

### Antibodies and western blotting

Antibodies used were as follows: anti-synaptophysin, anti-ChAT, Anti-PSD95, anti-NeuN (Cell Signaling Technology, Beverly, MA); anti-GPX4 (Santa Cruz Biotechnology, Dallas, TX); anti-4-HNE antibody (R&D Systems, Minneapolis, MN); and anti-β-actin antibody (Abcam, Cambridge, MA).

Tissues were homogenized in RIPA buffer (20 mM Tris, pH 7.4, 0.25 M NaCl, 1 mM EDTA, 0.5% NP-40, and 50 mM sodium fluoride) supplemented with protease inhibitors (539,134, EMD Biosciences Inc., San Diego, CA). Levels of specific proteins in tissues were determined by western blots as previously described^44^. In brief, 30 µg total protein per sample was separated by SDS-PAGE and transferred to PVDF membranes. Membranes were blocked with 5% BSA then incubated with primary antibody overnight at 4 °C. After incubation with fluorophore-conjugated secondary antibodies (ThermoFisher, MA) for 1 h, bands were detected using an Odyssey scanner (LI-COR, Lincoln, NE). Alternatively, after incubation with primary antibodies, the membranes were incubated with a HRP-conjugated secondary antibody. The bands were visualized using the ECL Kit (RPN2132, GE Healthcare, Piscataway, NJ). The bands were quantified using NIH ImageJ software (ImageJ 1.52a; http://imagej.nih.gov/ij) and normalized to the loading control (β-Actin). The experiments were repeated at least once.

### Spinal cord section preparation and staining

Mice were euthanized with CO_2_ inhalation followed by cervical dislocation. After dissecting out the spine, the spinal cord was flushed out with chilled saline using a blunt needle attached to a 5-mL syringe. The lumbar spinal cords were fixed in 4% paraformaldehyde at 4 °C overnight and were then equilibrated in 30% sucrose in PBS for 1–2 days at 4 °C. The spines were frozen by submersion in 2-metylbutane chilled in dry ice. The spine sections at a thickness of 16 µm were made using a cryostat.

For Nissl staining, sections were stained with 0.1% (w/v) cresyl violet for 5 min, dehydrated through graded ethanol rinses, and then cleared in xylene. For immunofluorescence staining, spinal cord sections were blocked with blocking buffer (1% horse serum in PBS) for 30 min and then incubated with primary antibodies in PBS at 4 °C overnight. The sections were then washed 3 times with PBS and incubated with the fluorophore-conjugated secondary antibody in PBS for 1 h at room temperature. After washing 3 times, slides were mounted with ProLong Gold Antifade Reagent (P36930, Invitrogen, Carlsbad, CA).

### Measurement of Gpx4 activity

Gpx4 Activity was measured using phosphatidylcholine hydroperoxide (PCOOH) as substrate^45^. To synthesize PCOOH, 5 mg of L-α-Phosphatidylcholine (Sigma-Aldrich, St. Louis, MO) was reacted with lipoxygenase (~ 250,000U) (Sigma-Aldrich, St. Louis, MO) in the presence of oxygen for 30 min. The reaction mixture (i.e., PCOOH mixture) was then applied to a Sep-Pak C18 Cartridge. After washing with 220 ml water, PCOOH was eluted with 2 ml Methanol. Methanolic solution of PCOOH was stored at -20 °C and used within 3 weeks.

Spinal cord tissues were homogenized in Lysis buffer (50 mM Tris–HCl [pH 7.5], 0.5 mM EDTA, 1.5% Triton X-100, 0.5 mM DTT, and 1 mM PMSF) supplemented with protease inhibitors. After centrifugation (10,000 × *g* for 5 min at 4 °C), supernatants were transferred to fresh tubes pre-chilled on ice, and the protein concentrations of supernatants were measured. Gpx4 activities in spinal cord extracts (i.e., supernatants) were measured in Reaction Buffer (0.1 m Tris–HCl [pH8.0), 2 mM EDTA, 1.3 mM NaN3, 0.1% Triton X-100) containing glutathione reductase (1.5 U/ml), glutathione (3 mM), and NADPH (0.2 mM). The reactions were initiated by quickly adding PCOOH (~ 0.3 mM). Absorbance at 340 nm was measured every 20 s over a 10–15 min period in a spectrophotometer. The Gpx4 activity in the spinal cord tissue was expressed as Units/mg protein, with one unit of Gpx4 defined as the oxidation of 1 nmol of NADPH to NADP^+^ per minute at 25 °C. The experiments were repeated at least once.

### Measurement of glutathione level

Total GSH levels in the spinal cord tissues were determined using a Glutathione Colorimetric Detection Kit (EIAGSHC, ThermoFisher, MA). Spinal tissue lysates were obtained, and the GSH assay was performed using protocols provided by the manufacturer.

### Real-time qRT-PCR

Real-Time qRT-PCR was conducted as described previously^46^. The mRNA levels were normalized to β-Actin to control for input RNA. Primers used were as follows: Gpx4 (forward: 5’-AGT ACA GGG GTT TCG TGT GC-3’; reverse: 5’-CAT GCA GAT CGA CTA GCT GAG -3’); hSOD1 (forward, 5’- GGT GGG CCA AAG GAT GAA GAG-3’; reverse, 5’- CCA CAA GCC AAA CGA CTT CC -3’); β-Actin (forward: 5’-ATC TGG CAC CAC ACC TTC TAC-3’; reverse: 5’-CAG GTC CAG ACG CAG GAT G-3’).

### Plasmid constructs and cell procedures

Plasmid constructs expressing human WT SOD1 (SOD1^WT^) and human SOD1 with G93A mutation (SOD1^G93A^) originally generated by the Fisher lab were obtained through Addgene^20^. The GPX4 expression construct was generated in-house in which the expression of human Gpx4 is directed by the CAG promoter.

NSC-34 cell line was purchased from Cedarlane Co. (Burlington, Ontario, Canada). NSC-34 cells were cultured in DMEM medium supplemented with 10% FBS and 1 × penicillin/streptomycin in 5% CO2, 95% air at 37 ˚C. The cells were passaged every 2–3 days until use. Ferrostatin-1 (Cat# S7243), Liproxstatin-1 (Cat #-S7699) and Erastin (Cat# S7242) were purchased from Selleckem.com. BODIPY581/591 C11 was purchased from ThermoFisher Scientific (Waltham, MA).

For the transfection study, NSC-34 cells were plated into a 24-well plate at 2 × 10^5^ cells per well. On the next day, cells were transfected with SOD1^WT^ (1 ug) or SOD1^G93A^ (1 ug) using transfection reagent Lipofectamine 2000 (ThermoFisher Scientific, Waltham, MA). For co-transfection, NSC34 cells were transfected with SOD1^G93A^ (1 ug) construct plus GPX4 expression construct (1 ug) or with SOD1^G93A^ construct (1 ug) plus vector construct (1 ug). After transfection, cells were cultured in DMEM medium without FBS.

C11-BODIPY flow cytometry. NSC-34 cells were transfected with either SOD1^WT^ or SOD1^G93A^ plasmids. Forty-eight hours after transfection, cells were gently washed using Hank’s Balanced Salt Solution (HBSS), and stained using 2.5 μM C11-BODIPY in HBSS for 30 min. After staining, cells were washed and trypsinized. Cells were then pelleted from solution, and washed/pelleted again before resuspending in HBSS. Resuspended cells were filtered, and immediately subject to flow analysis. The experiments were repeated 3 times.

Cell survival determination. 72 h after transfection, cells were harvested by trypsin digestion and re-suspended in 100 uL of 1 × PBS. 10 ul of cell suspension was mixed gently with 10 ul trypan blue stain, the sample mixture was then added to the chamber ports of the counting slide, and the numbers of alive and death cells were recorded using Countess Automated Cell Counter (Invitrogen, Carlsbad, CA). The experiments were repeated 3 times.

### Statistics

Data are expressed as mean ± SEM. Results were statistically analyzed using two-way ANOVA or Student’s t-test when appropriate. Statistical significance was set to a minimum of *p* < 0.05. Lifespan curves of mice were compared by The log rank test using GraphPad Prism version 7.05 (GraphPad Software, San Diego, CA).

## Supplementary Information

Supplementary Information.
