# Supplementary material for: Overexpression of ferroptosis defense enzyme Gpx4 retards motor neuron disease of SOD1G93A mice
Source: Sci Rep. 2021 Jun 18;11:12890. doi: 10.1038/s41598-021-92369-8 (PMC8213805; doi:10.1038/s41598-021-92369-8)
Supplement: Supplementary file 1 — Supplementary Information. [file 41598_2021_92369_MOESM1_ESM.pdf]

Title:

Overexpression of ferroptosis defense enzyme Gpx4 retards motor neuron disease of SOD1G93A mice

Authors: Liuji Chen, Ren Na, Kirsten McLane, Cody Sylvester Thompson, Ju Gao, Xinglong Wang, Qitao Ran

Supplementary Material

One supplemental Table and ten supplemental figures are provided to give additional information for the study.

Case ID	Age (Year)	PMI (hour)	Diagnosis	Gender
A1	40	15.6	ALS	M
A2	54	29.1	ALS	M
A3	59	20.3	ALS	F
A4	61	7.5	ALS	F
A5	70	18.9	ALS	M
A6	76	8.6	ALS	F
A7	81	4	ALS	F
A8	81	17.2	ALS	M
A9	82	20.5	ALS	M
A10	82	12.1	ALS	F
C1	41	13	control	M
C2	41	20	control	M
C3	41	14	control	M
C4	42	27	control	F
C5	45	19	control	M
C6	49	26	control	F
C7	50	15	control	F
C8	50	24	control	M
C9	65	25	control	M
C10	74	18	control	M

Supplemental Table 1. Information of human lumber spinal cord tissues used in this study.

s1A

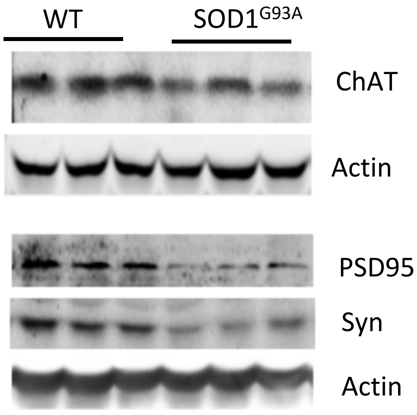

s1B

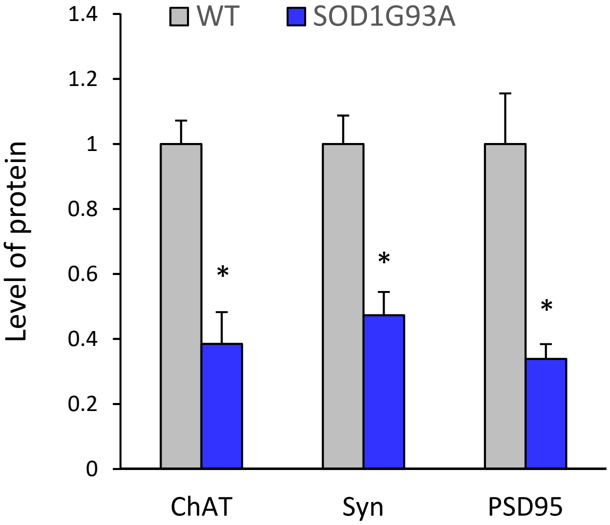

Supplemental Fig. 1. Loss of neuronal proteins in advanced symptomatic stage in SOD1^{G93A} mice. s1A. Graph of western blots showing levels of neuronal marker proteins in lumbar spinal cord tissues of WT and SOD1^{G93A} mice at 150 days of age. Syn: synaptophysin. **s1B**. Quantified results of western blots. n = 3. *: $p < 0.05$.

s2A

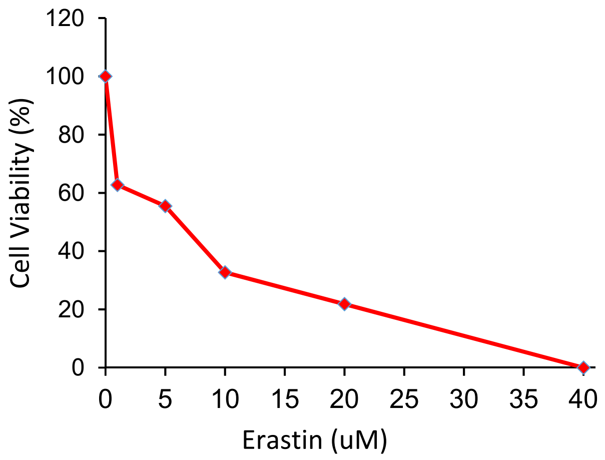

s2B

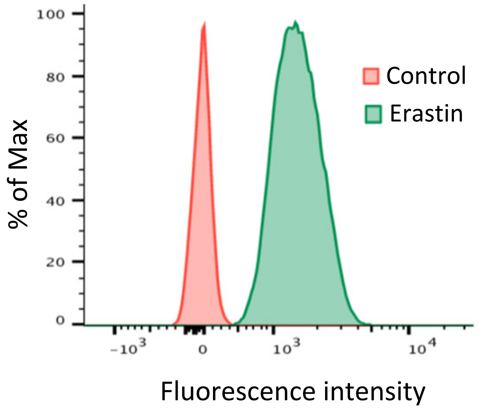

Supplemental Fig. 2. Erastin treatment induced cell death in NSC-34 cells as well as increased lipid ROS. s2A. Viabilities of NSC-34 cells exposed to Erastin. **s2B.** Graph of flow cytometry analysis showing oxidation of BODIPY581/591 C11 in NSC-34 cells exposed to 10 μM Erastin

s3A

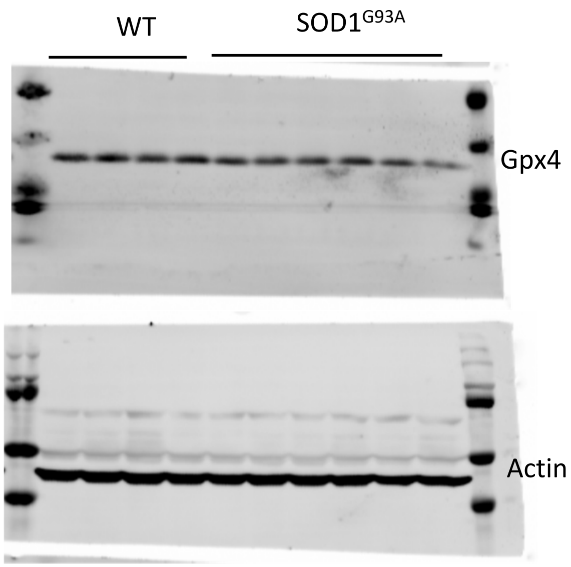

s3B

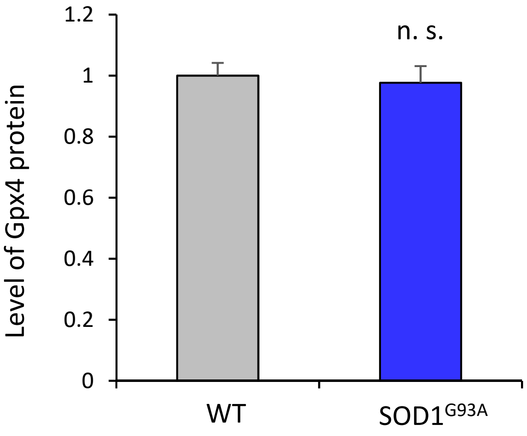

Supplemental Fig. 3. Level of Gpx4 protein in the spinal cords of asymptomatic SOD1^{G93A} mice. s3A. Graph of western blots showing levels of Gpx4 proteins in lumbar spinal cord tissues of WT and asymptomatic SOD1^{G93A} mice (60 days of age). **s3B**. Quantified results of western blots. n. s.: not statistically significant

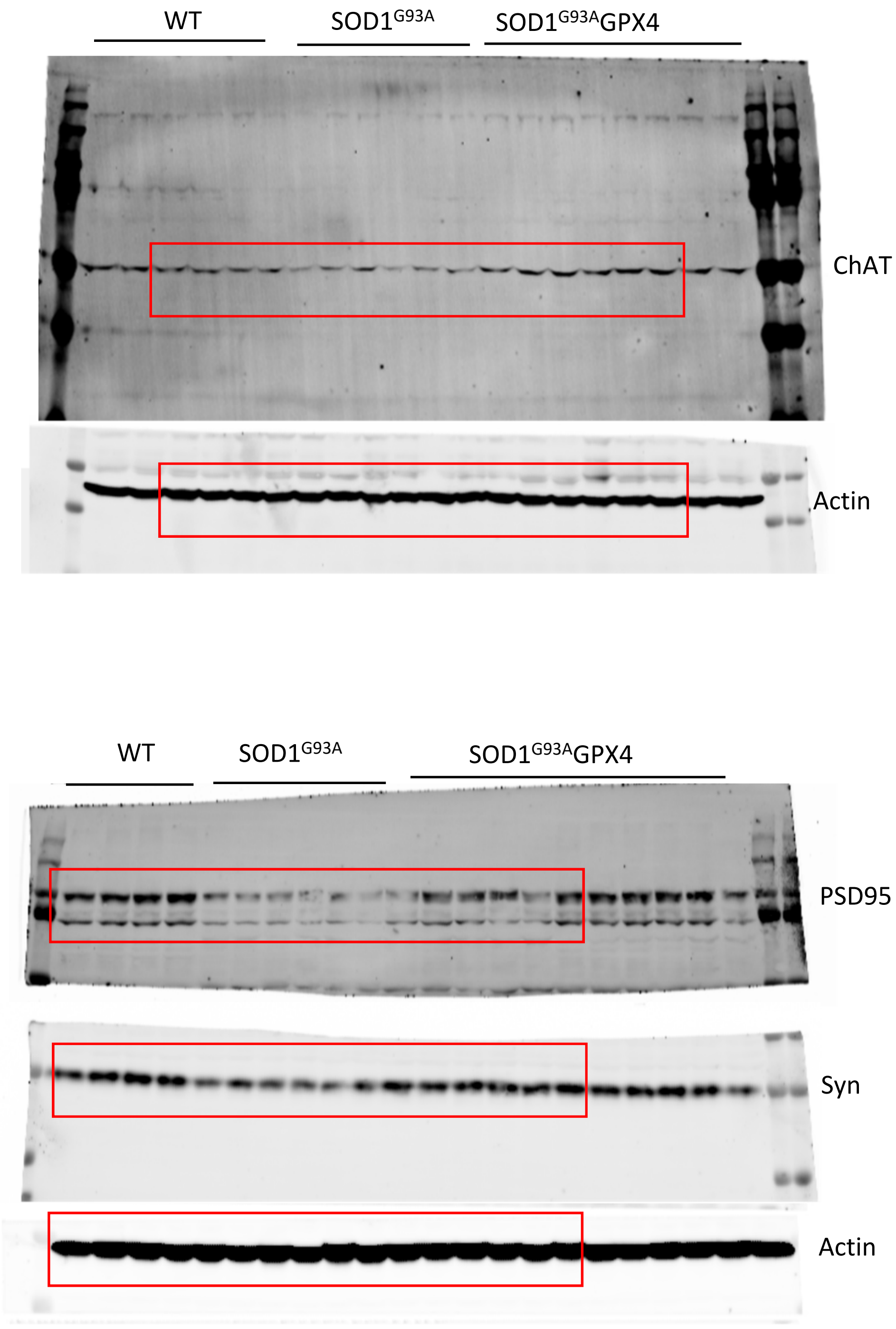

Supplemental Fig. 4. Full western blots of Fig 4A. Red boxes indicate regions of blots used**.**

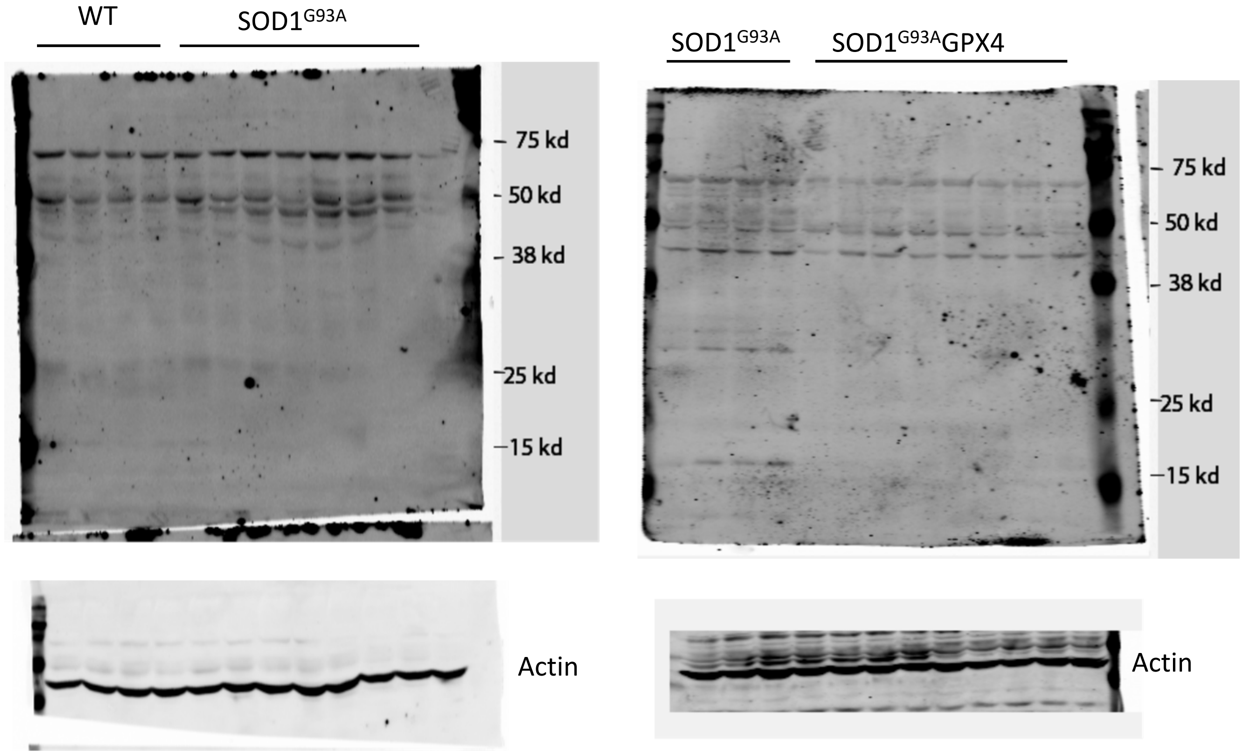

Supplemental Fig. 5. 4HNE blots for Fig. 4D

s6A

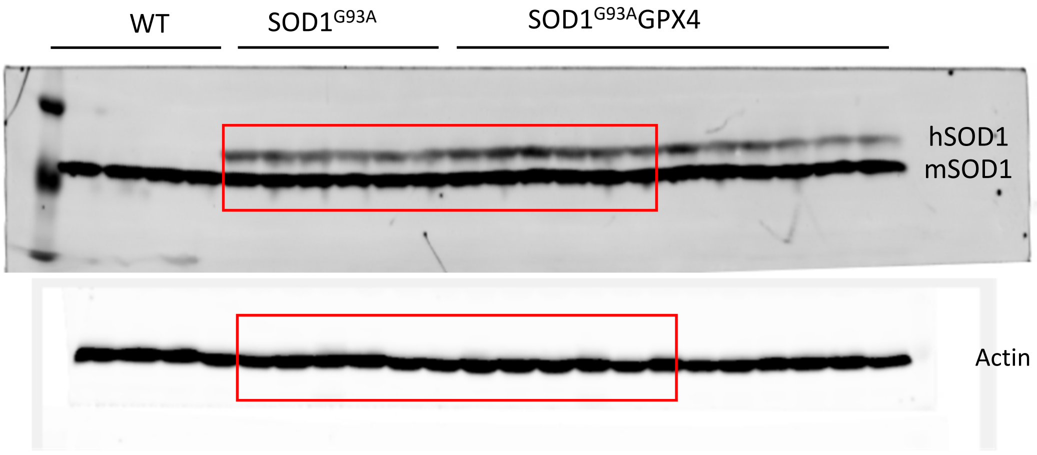

s6B

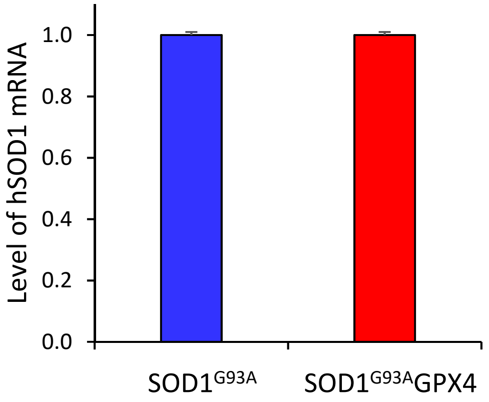

Supplemental Fig. 6. A. Full western blots of Fig 4E. Red boxes indicate regions of blots used**. B.** Levels of hSOD1 mRNA quantified by qRT-PCR. n = 4.

s7A

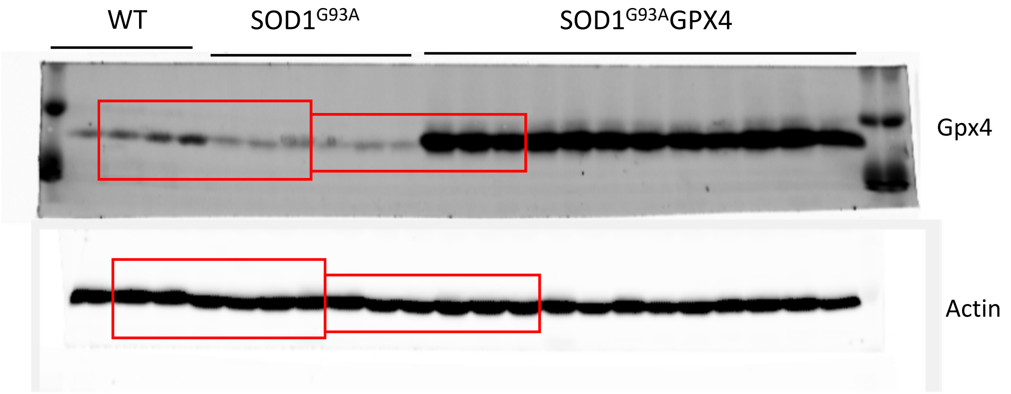

s7B

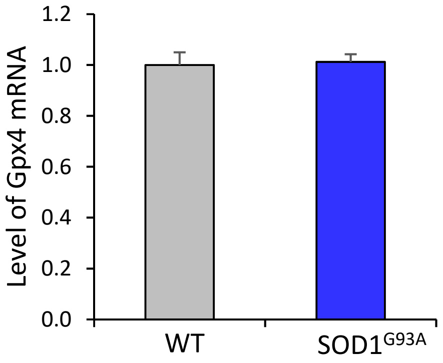

Supplemental Fig. 7. s7 A. Western blots showing levels of Gpx4 protein in spinal tissues of WT, symptomatic SOD1^{G93A} mice and SOD1^{G93A} mice (120 days of age). Small and big red boxes indicate regions of blots used in Fig. 1B and Fig. 6A, respectively. **s7B.** Levels of Gpx4 mRNA quantified by Real-Time qRT-PCR.

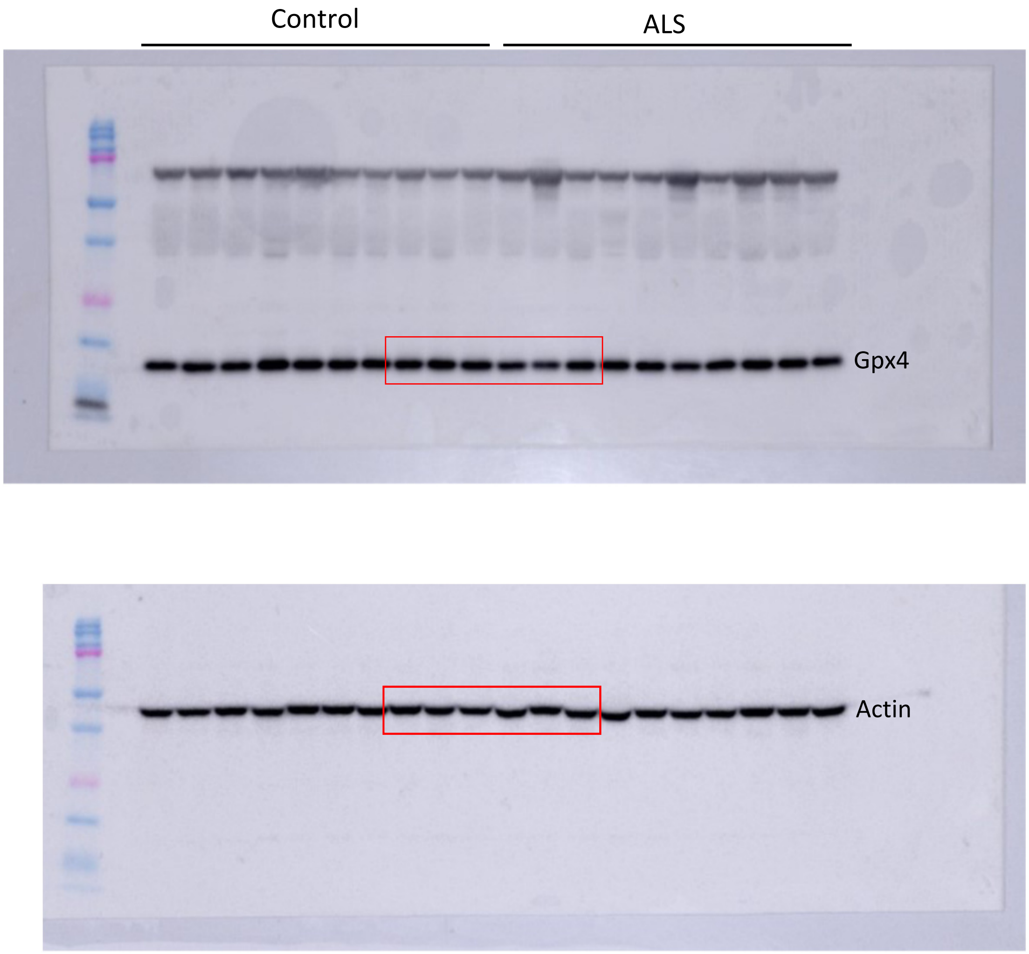

Supplemental Fig. 8. Western blots showing levels of Gpx4 protein in spinal tissues from ALS cases and Control (non-ALS) cases. Red boxes indicate regions of blots used in Fig. 6F.

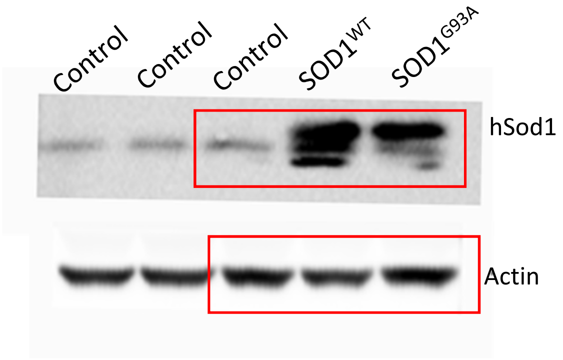

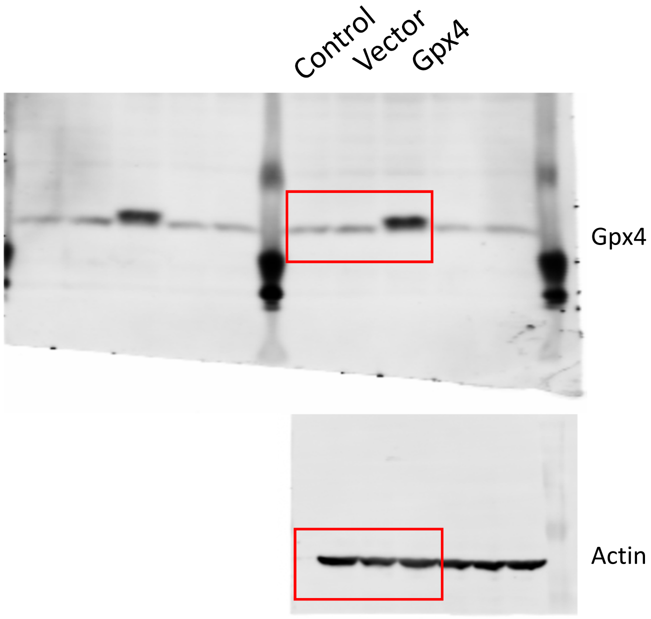

Supplemental Fig. 9. Western blots of Fig. 5A and Fig. 5B. Red boxes indicate regions of blots used.

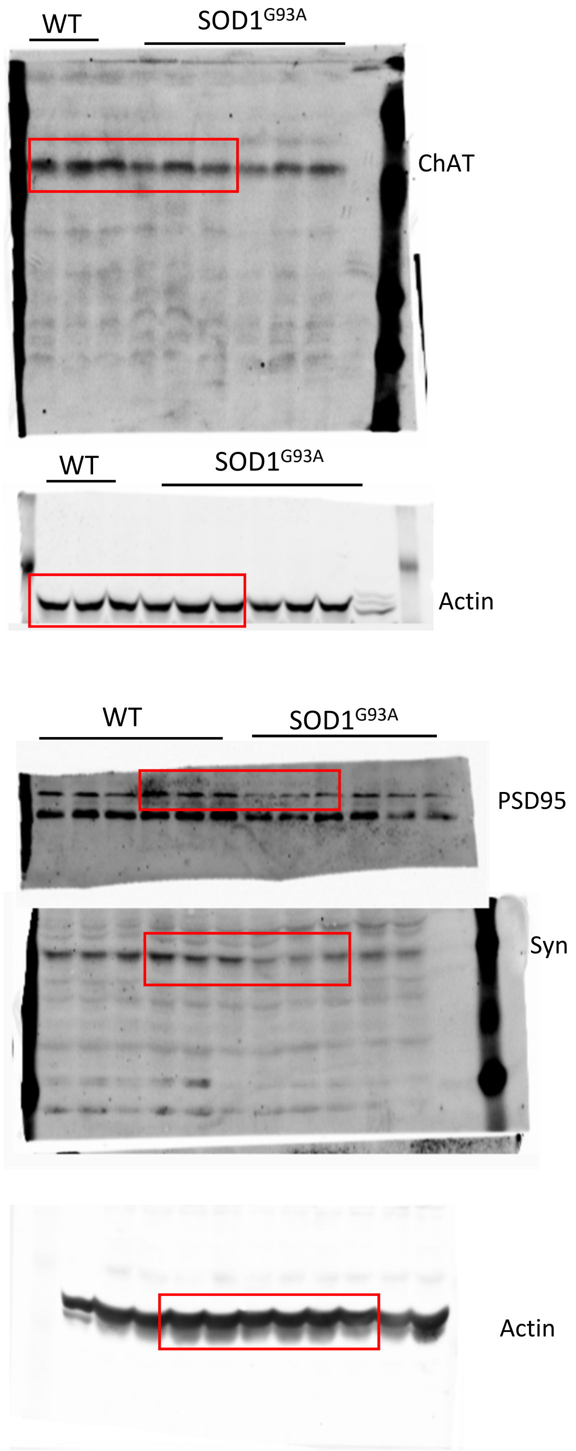

Supplemental Fig. 10. Western blots of Supplemental Fig. 1A. Red boxes indicate regions of blots used.
